# Inhibition of K_Ca_2 Channels Decreased the Risk of Ventricular Arrhythmia in the Guinea Pig Heart During Induced Hypokalemia

**DOI:** 10.3389/fphar.2020.00749

**Published:** 2020-05-20

**Authors:** Jonas Goldin Diness, Lea Abildgaard, Sofia Hammami Bomholtz, Mark Alexander Skarsfeldt, Nils Edvardsson, Ulrik S. Sørensen, Morten Grunnet, Bo Hjorth Bentzen

**Affiliations:** ^1^Acesion Pharma, Copenhagen, Denmark; ^2^Department of Biomedical Sciences, Faculty of Health and Medical Sciences, University of Copenhagen, Copenhagen, Denmark; ^3^Department of Molecular and Clinical Medicine/Cardiology, Institute of Medicine, Sahlgrenska Academy, University of Gothenburg, Gothenburg, Sweden

**Keywords:** K_Ca_2 channel inhibitor, hypokalemia, dofetilide, Kv11.1 blockers, SK channel, SK channel inhibitor, arrhythmia, antiarrhythmic drug

## Abstract

**Background:**

Hypokalemia reduces the cardiac repolarization reserve. This prolongs the QT-interval and increases the risk of ventricular arrhythmia; a risk that is exacerbated by administration of classical class 3 anti-arrhythmic agents.

Small conductance Ca^2+^-activated K^+^-channels (K_Ca_2) are a promising new atrial selective target for treatment of atrial fibrillation. Under physiological conditions K_Ca_2 plays a minor role in ventricular repolarization. However, this might change under hypokalemia because of concomitant increases in ventriculay -60r intracellur Ca^2+^.

**Purpose:**

To study the effects of pharmacological K_Ca_2 channel inhibition by the compounds AP14145, ICA, or AP30663 under hypokalemic conditions as compared to dofetilide and hypokalemia alone time-matched controls (TMC).

**Methods:**

The current at +10 mV was compared in HEK293 cells stably expressing K_Ca_2.3 perfused first with normo- and then hypokalemic solutions (4 mM K^+^ and 2.5 mM K^+^, respectively). Guinea pig hearts were isolated and perfused with normokalemic (4 mM K^+^) Krebs-Henseleit solution, followed by perfusion with drug or vehicle control. The perfusion was then changed to hypokalemic solution (2.5 mM K^+^) in presence of drug. 30 animals were randomly assigned to 5 groups: ICA, AP14145, AP30663, dofetilide, or TMC. QT-interval, the interval from the peak to the end of the T wave (Tp–Te), ventricular effective refractory period (VERP), arrhythmia score, and ventricular fibrillation (VF) incidence were recorded.

**Results:**

Hypokalemia slightly increased K_Ca_2.3 current compared to normokalemia. Application of K_Ca_2 channel inhibitors and dofetilide prolonged the QT interval corrected for heart rate. Dofetilide, but none of the K_Ca_2 channel inhibitors increased Tp–Te during hypokalemia. During hypokalemia 4/6 hearts in the TMC group developed VF (two spontaneously, two by S1S2 stimulation) whereas 5/6 hearts developed VF in the dofetilide group (two spontaneously, three by S1S2 stimulation). In comparison, 0/6, 1/6, and 1/6 hearts developed VF when treated with the K_Ca_2 channel inhibitors AP30663, ICA, or AP14145, respectively.

**Conclusion:**

Hypokalemia was associated with an increased incidence of VF, an effect that also seen in the presence of dofetilide. In comparison, the structurally and functionally different K_Ca_2 channel inhibitors, ICA, AP14145, and AP30663 protected the heart from hypokalemia induced VF. These results support that K_Ca_2 inhibition may be associated with a better safety and tolerability profile than dofetilide.

## Introduction

Intra- and extra-cellular K^+^ concentrations are closely regulated physiologically. Hypokalemia, defined as K^+^ serum levels below 3.5 mM, has been reported to occur in 16% of patients when first admitted to acute medical departments (Jensen et al., 2015). Hypokalemia is an adverse effect of diuretic therapy prescribed to treat hypertension, heart failure and other conditions, but can also develop from e.g. vomiting and diarrhea. Hypokalemia lowers the Na/K-ATPase activity, hyperpolarizes the resting membrane potential, and despite increasing the driving force for outward K^+^ current it suppresses the cardiac I_K1_, I_Kr_, and I_to_ currents. This reduction in the repolarizing reserve in turn prolongs the cardiac action potential, and promotes the development of early-after depolarizations (EAD) and initiation of ventricular tachycardia (VT) (for review see (Weiss et al., 2017)). Moreover, action potential prolongation and hypokalemia increase intracellular Ca^2+^ loading through increased Ca^2+^ influx *via* calcium channels, and by lowered Na^+^/Ca^2+^ activity (calcium efflux) secondary to the reduced Na^+^/K^+^-ATPase activity and consequent elevated intracellular Na^+^ concentrations (Aronsen et al., 2015; Weiss et al., 2017). It has been suggested that the increase in intracellular calcium activates ventricular K_Ca_2 channels, functioning as a protective mechanism against ventricular arrhythmia during hypokalemia (Chan et al., 2015). If that is the case, K_Ca_2 channel inhibition should be proarrhythmic under hypokalemic conditions. The K_Ca_2 channel also known as the small conductance calcium activated K^+^, or SK, channel is a novel drug target for treatment of atrial fibrillation (AF) (Diness et al., 2010; Qi et al., 2014; Skibsbye et al., 2014; Haugaard et al., 2015; Diness et al., 2017). Under physiological conditions K_Ca_2 channels appear to play a minor role in ventricular repolarization. However, this might change under pathophysiological conditions such as heart failure and myocardial infarction or hypokalemia (Chua et al., 2011; Chang et al., 2013a; Chang et al., 2013b; Gui et al., 2013; Bonilla et al., 2014; Chan et al., 2015; Hundahl et al., 2017).

Classical class III anti-arrhythmic drugs such as sotalol and dofetilide inhibit K_V_11.1 thereby reducing I_Kr_ and prolonging the QT-interval (Redfern et al., 2003). The drug-induced impairment of the repolarizing reserve and consequent risk for ventricular arrhythmia is further potentiated by hypokalemia (McKibbin et al., 1984).

Because hypokalemia increases intracellular calcium and compromises the repolarizing reserve, we hypothesized that inhibition of K_V_11.1 and K_Ca_2 would be pro-arrhythmic in a hypokalemic setting. To study this we investigated the effects of K_Ca_2 channel inhibition under hypokalemic conditions as compared to the class III anti-arrhythmic agent dofetilide. Moreover, we also explored if K_Ca_2.3 channel conductance per se is affected by hypokalemia.

## Materials and Methods

### Electrophysiology

The experiments were performed on HEK293 cells stably expressing K_Ca_2.3. The cells were cultured in Dulbecco’s modified Eagle’s medium (DMEM1965, Substrat- og sterilcentralen, University of Copenhagen, Denmark) supplemented with 10% fetal bovine serum (Biowest, France), 100 U/ml of penicillin/streptomycin (Sigma, Germany), and 100 µg/ml geneticin (Gibco, USA).Whole cell patch clamping was performed on an automated whole-cell patch-clamp system (QPatch 16 HT) with single-hole Qplates (Sophion, Denmark). The Qpatch automatically generates giga seals, whole-cell formation, compound application, voltage-clamping, and recording of current. On the day of experiment, HEK293 cells expressing human K_Ca_2.3 were treated with detachin (Genlantis, CA, USA) and resuspended in serum Free medium (C5467 SAFC, Buchs, Switzerland) containing 25 mM HEPES 0.04 mg/ml soy bean trypsin inhibitor (T6522 Sigma) and 100U/ml penicillin/streptomycin.

The extracellular solution consisted of (in mM): NaCl 145; CaCl_2_ 2; MgCl_2_ 1; 10 HEPES and 10 glucose and 4 mM KCl or 2.5 mM KCl for the normo- and hypokalemic conditions. The pH was adjusted to 7.4 with NaOH. The intracellular solution contained (in mM): 154 KCl, 10 mM HEPES and 10 mM EGTA. CaCl_2_ was added to give calculated free concentrations of Ca^2+^ of 0.4 µM (K0.4) and MgCl_2_ was added to give a free concentration of 1 mM. Free concentrations were calculated by the Eqcal software.

Currents were elicited by a voltage ramp from −140 mV to +20 mV (200 ms in duration) applied every fifth second from a holding potential of −80 mV. Data were sampled at 10 kHz, four-order Bessel filter, cutoff frequency 3 kHz, and 80 % Rs compensation. The currents were recorded for 500 s in normokalemic solution before exchanging to hypokalemic extracellular solution for 200 s.

### Isolated Perfused Guinea Pig Heart Experiments

Guinea pig experiments were performed under licenses from the Danish Ministry of justice (2018-15-0201-01430 and 2016-15-0201-00850) and in accordance with the Danish guidelines for animal experiments according to the European Commission Directive 86/609/EEC.

30 female Guinea pigs (weighing between 350 and 600 g) from the Dunkin Hartley strain (HsdPoc : DH) (Charles River, Scanbur A/S, Karlslunde, Denmark) were used for the study, and randomized to a hypokalemia alone time-matched control group (TMC) or groups of n = 6 treated with ICA, AP14145, AP30663, or dofetilide. The animals were anaesthetized (200 mg/kg pentobarbital and lidocaine hydrochloride 0.150 ml/100 g body weight i.p., (Glostrup Apotek, Denmark)), and a dose of 1000 IU/kg heparin was injected intravenously. After artificial ventilation was established (volume, 12 ml/kg; rate, 60 strokes/min) a thoracotomy was performed. The perfusion cannula was inserted and fixed in the aorta for retrograde perfusion with (normokalemic) Krebs-Henseleit solution: (in mM L^−1^: NaCl 120.0, NaHCO_3_ 25.0, KCl 4.0, MgSO_4_ 0.6, NaH_2_PO_4_ 0.6, CaCl_2_ 2.5, glucose 11.0, saturated with 95% O_2_ and 5% CO_2_, 37°C, pH 7.4). Hearts were then mounted in a Langendorff set-up (Hugo Sachs Elektronik, Harvard Apparatus GmbH, Germany) and perfused at a constant perfusion pressure of 60 mmHg. Electrocardiography (ECG) was measured with three ECG electrodes placed close to the heart. A pacing electrode was placed on the left ventricle. All data were acquired at 2 KHz using the 16-channel PowerLab system (ADInstruments, Oxford, UK), and monitoried by LabChart 7 software (ADInstruments).

**Figure 1:**
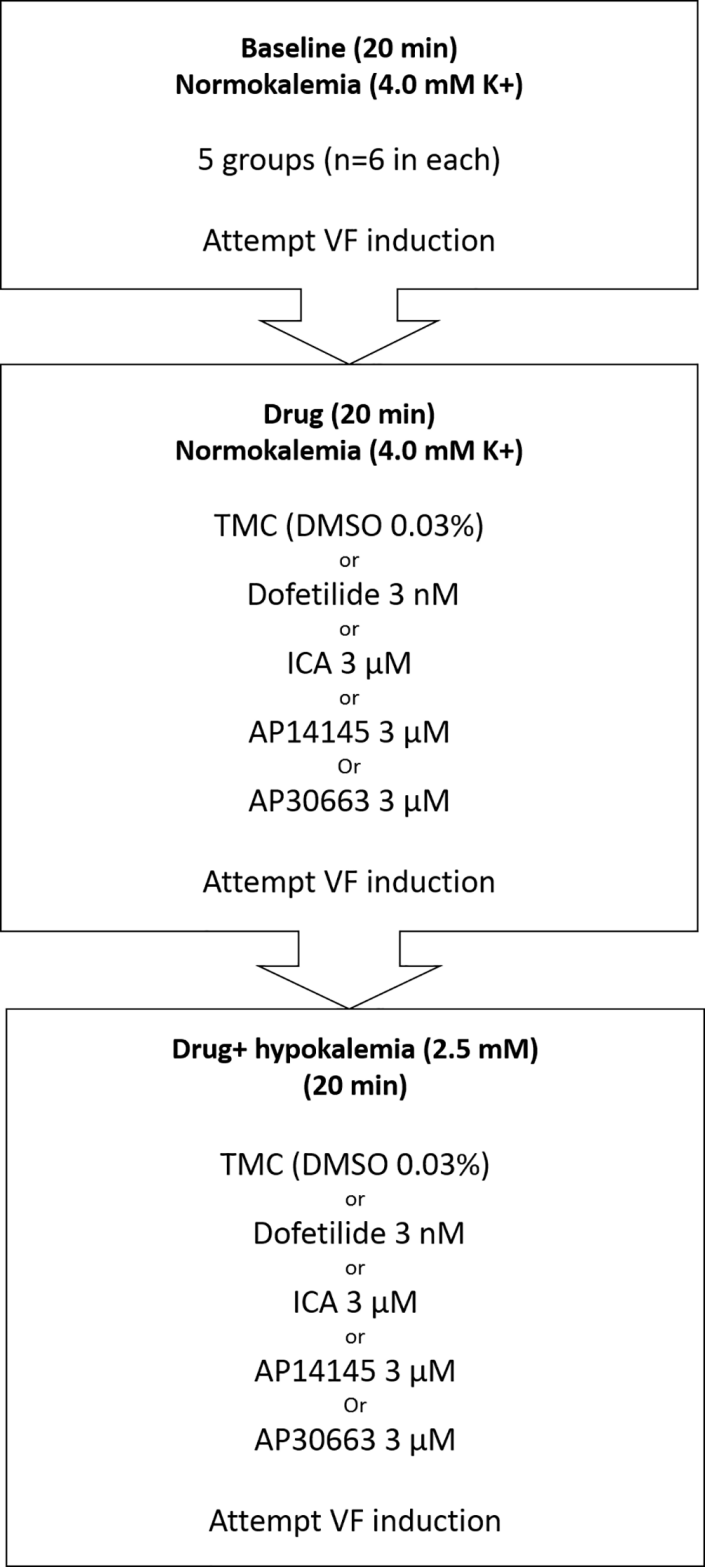
Flowchart of isolated perfused guinea pig experiments and the five experimental groups (TMC, Dofetilide, ICA, AP14145, and AP30663). Three 20-min perfusion periods: baseline, drug, and drug + hypokalemia. VF induction by electrical stimulation (S1–S2) was attempted at the end of each period. TMC, time-matched control; VF, ventricular fibrillation.

After stabilization, the heart was subjected to three subsequent 20-min perfusion periods (**Figure 1**): 1) baseline recording in normokalemia (4 mM K^+^), 2) drug (ICA, AP14145, AP30663, dofetilide, or DMSO) + normokalemia, and 3) drug + hypokalemia (2.5 mM K^+^). At the end of each perfusion period ventricular effective refractory period (VERP) was measured by applying electrical stimulation (five times rheobase) with a fixed interval of 200 ms (S1S1 stimulation) and for every 10^th^ beat an extra stimulus (S2) was applied with 10 ms decrements starting from 150 ms. The VERP was defined as the longest S1–S2 interval failing to elicit a ventricular action potential. Between the VERP recordings, the heart was kept unpaced. QT-intervals and the intrinsic heart rate (HR) were analyzed just prior to electrical stimulation (mean of 100 last beats).

### Arrhythmia Scoring

The ECG recordings from the three perfusion periods were analyzed for the incidence of ventricular extra systoles (VES) and occurrence of bi-, tri-, and quadrigeminy, VT, and ventricular fibrillation (VF). VT was defined as six or more consecutive VES with an increased HR of >50 beats per minute (bpm). Non-sustained VT (NSVT) < 30 beats; sustained VT (SVT) > 30 beats.

A predefined arrhythmia score system was used to score the severity of the arrhythmia see **Table 1**. The arrhythmia score was the sum of the individual scores.

**Table 1:** Arrhythmia scoring.

Arrhythmia	Score
<5 ventricular extra systoles (VES)	0
5-25 VES	1
>25 VES	2
Bigeminy	3
Non-sustained ventricular tachycardia (NSVT)	4
Sustained ventricular tachycardia (SVT)	5
Induced ventricular fibrillation (VF)	10
Spontaneous VF	15

The number of VES was counted during the last unpaced 5 min of perfusion period one and two and during the last 15 min of hypokalemia + drug (T45–T60). The occurrence of bi-, or tri-, or quadri-geminy, NSVT, SVT, induced and spontaneous VF were counted during the entire perfusion period. Induced VF refers to when VF was induced by incremental S1S2 stimulation during VERP determination.

### Data Analysis

All data and figures were analyzed using Prism 8 (GraphPad Software, CA, USA).

*In vitro* electrophysiology data were analyzed as follows: After removing outliers by ROUT testing (Q = 10%), a paired Wilcoxon test was used to compare the currents during normo- and hypokalemia.

QT-intervals were corrected for HR using the guinea pig specific HR QT correction formula: QTcH = QT/(RR/0.28)^0.7861^ (Holzgrefe et al., 2014).

Changes from baseline values were compared between each drug group with the TMC group for HR, QTcH, the interval from the peak to the end of the T wave (Tp–Te), VERP, and arrhythmia score by using two-way ANOVA or, in case of missing values, a mixed-effects analysis with Sidak’s multiple comparisons *post hoc* test. P values are given with three decimals.

## Results

### Effects of Hypokalemia on outward K^+^ K_Ca_2.3 Currents

**Figure 2:**
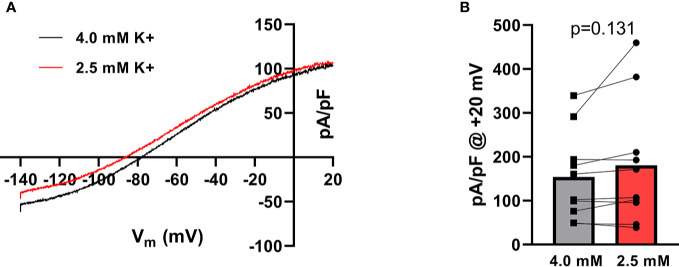
Hypokalemia shifts the reversal potential of K^+^ in HEK cells expressing K_Ca_2.3. **(A)** Current–voltage relationship of K_Ca_2.3 current measured at 4.0 mM extracellular K^+^ and at 2.5 mM K^+^. Currents were elicited by a voltage-ramp protocol from −140 to +20 mV (200 ms). **(B)** Summary graph showing the individual current levels at 2.5 mM and 4.0 mM K^+^, n = 10. Bars represent the mean values.

To investigate whether there was a direct effect on K_Ca_2.3 current as a consequence of change in extracellular K^+^ a number of patch-clamp experiments on heterologously expressed K_Ca_2.3 channels were conducted. As expected from the Nernst equation, when switching from 4 mM to 2.5 mM extracellular K+, the voltage at 0 current was shifted toward more hyperpolarized potentials (from −80.23 ± 1.22 to −87.13 ± 1.75 mV, p < 0.001, n = 10) (**Figure 2A**), and based on this, a higher driving force for K^+^-efflux from the cell at +20 mV is achieved. There was slightly more current at +20 mV during hypokalemia than during normokalemia (181 ± 44 pA/pF vs 154 ± 32 pA/pF, **Figure 2B**).

### Effects of Hypokalemia on Repolarization, Heart Rate, and Ventricular Refractoriness

**Figure 3:**
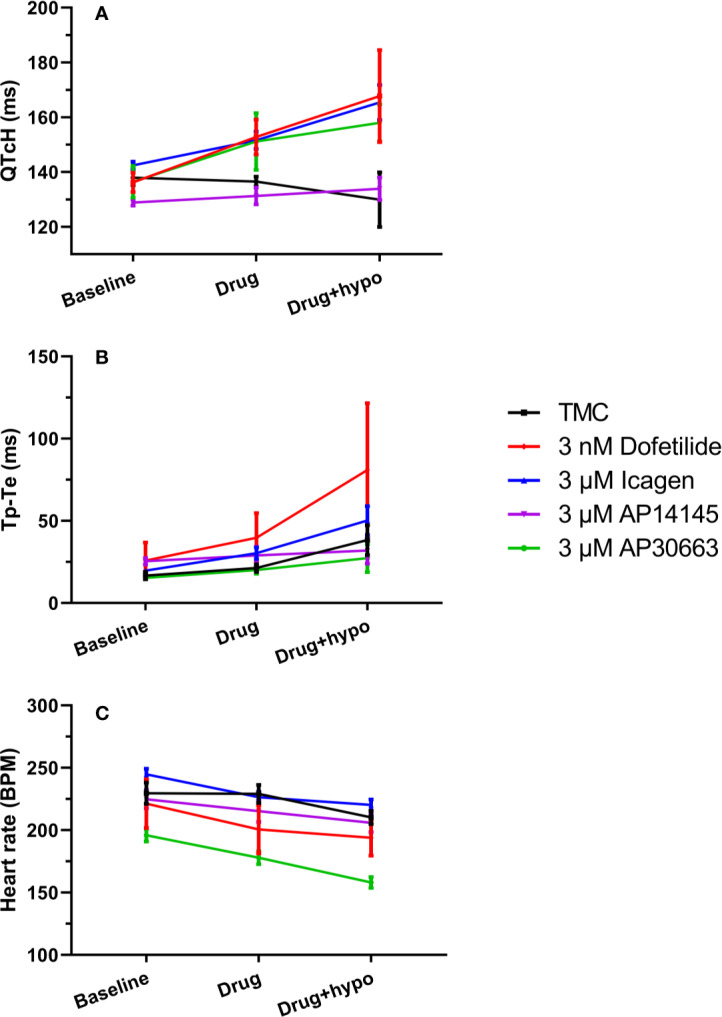
K_Ca_2 channel inhibitors prolong QTc and Tp-Te intervals, and decrease heart rate to a variable degree. **(A)** Changes in the QT interval corrected for heart rate (QTcH) **(B)** Changes ins the Tp-Te interval and **(C)** heart rate in the five groups. Data are presented as mean ± SEM. For QTc and heart rate: N = 6 except for measurement during hypokalemia where n = 5 for ICA and n = 4 for TMC and dofetilide because of spontaneous ventricular fibrillation. TMC, time-matched control.

A total of 30 animals were included in the study and randomly assigned to 5 groups: ICA, AP14145, AP30663, dofetilide, or TMC. Despite randomization there were baseline differences in QTcH and HR between the groups (**Figure 3**). Therefore we evaluated the effect of drugs by comparing changes from baseline (Δ-change = drug-baseline) between TMC and treatment groups for both perfusion periods (drug and drug + hypokalemia) (**Table 2**).

**Table 2:** Changes (Δdrug − baseline; Δdrug + hypo − baseline) in QTcH, and HR for each group.

	Drug/vehicle Δ-QTcH	n; p-value	Drug/vehicle + hypo Δ-QTcH		Drug Δ-HR	n, p-value	Drug/vehicle + hypo Δ-HR	n; p-value	Drug Δ-Tp–Te	n, p-value	Drug/vehicle+hypo Δ-Tp–Te	n; p-value
TMC	−1 ± 2	6; NA	−5 ± 7		−1 ± 2	6; NA	−9 ± 3	4; NA	5 ± 3	6; NA	21 ± 7	6; NA
3 nM Dofetilide	17 ± 3	6; 0.016	32 ± 11		−21 ± 5	6; 0.017	−29 ± 7	4; 0.055	14 ± 5	6; 0.725	57 ± 18	4; 0,015
3 µM ICA	9 ± 3	6; 0.250	24 ± 6		−18 ± 3	6; 0.040	−23 ± 8	5; 0.265	11 ± 4	6; 0.925	31 ± 9	5; 0,766
3 µM AP14145	2 ± 3	6; 0.921	5 ± 4		−10 ± 7	6; 0.480	−19 ± 5	6; 0.537	4 ± 5	6; > 0.999	6 ± 8	6; 0,434
3 µM AP30663	15 ± 3	6; 0.036	21 ± 3		−18 ± 3	6; 0.044	−38 ± 4	6; 0.001	5 ± 2	6; > 0.999	12 ± 9	6; 0,777

P-values refer to the comparison of Δ-values between TMC and treatment groups.

QTcH, QT interval corrected for heart rate; HR, heart rate; Tp–Te, the interval from the peak to the end of the T wave.

**Figure 4:**
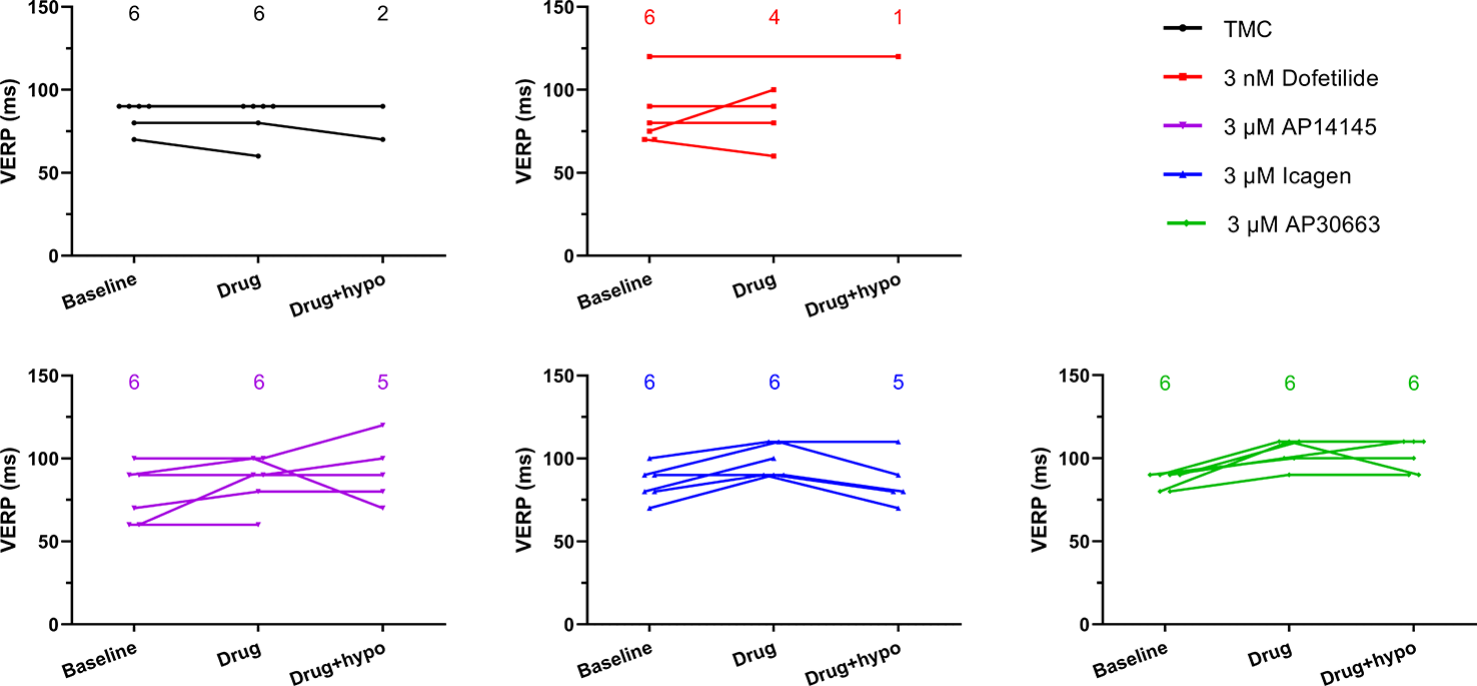
The ventricular effective refractory period (VERP) in the hypokalemia alone time-matched control (TMC) group as well as in the dofetilide group and three K_Ca_2 channel inhibitor groups at three time points. The number of data points for each time point are given in each graph. VERP could not be measured in all cases during hypokalemia due to the presence of ventricular fibrillation.

The effects of hypokalemia (2.5 mM K^+^) versus normokalemia (4.0 mM K^+^) were studied in the TMC group. The QTcH at baseline was 138 ± 3 ms during normokalemia and 130 ± 10 ms during hypokalemia (**Figure 3A**, **Table 2**). There was also no effect on HR (**Figure 3B**, **Table 2**). While no effects on QT and VERP were observed, the dispersion of ventricular repolarization marked by the Tp–Te interval was increased from 17 ± 2 ms during baseline to 38 ± 9 ms during hypokalemia (**Figure 3**, **Tables 2** and **3**) and a ventricular impact of hypokalemia was clearly visible by the increased incidence of arrhythmia (**Figure 4**). The small impact of hypokalemia on QT and the increased arrhythmia susceptibility reflect what has been previously reported in guinea pigs (Osadchii and Olesen, 2009).

### Effects of K_v_11.1 or K_Ca_2 Inhibition on Repolarization, HR, and Ventricular Refractoriness

During normokalemia, dofetilide and AP30663 both increased QTcH versus baseline compared with TMC (**Table 2**, **Figure 3A**). The K_Ca_2 channel inhibitor ICA increased QTcH versus baseline, but not in a statistically significant manner compared to TMC. However, none of the K_Ca_2 channel inhibitors nor dofetilide affected the Tp–Te interval during normokalemia compared to the TMC group (**Table 2**).

Dofetilide, AP30663, and ICA all decreased HR versus baseline compared with TMC (**Table 2**, **Figure 3B**).

The VERP at a paced HR of 300 bpm, was increased by the K_Ca_2 channel inhibitors AP30663 and ICA, but not by the K_v_11.1 blocker dofetilide. The K_Ca_2 channel inhibitor AP14145 did not significantly change QTcH, VERP, or HR versus baseline when compared to TMC.

### Effects of K_Ca_2 or K_v_11.1 Inhibition on Ventricular Refractoriness and Repolarization and on HR During Hypokalemia

During hypokalemia dofetilide, AP30663, and ICA all continued to increase QTcH versus baseline compared with TMC (**Table 2**, **Figure 3A**). Interestingly, dofetilide increased the dispersion of ventricular repolarization during hypokalemia even more than hypokalemia alone; the Tp–Te increase from baseline was 57 ± 18 ms compared to the increase seen in the TMC group which was increased by 21 ± 7 ms by hypokalemia. None of the K_Ca_2 channel inhibitors affected Tp–Te during hypokalemia compared to the TMC group.

In the TMC group the HR was decreased during hypokalemia, and the decrease in HR versus baseline seen by dofetilide and ICA during hypokalemia was no longer significantly different from the decrease of HR in the TMC group during hypokalemia (**Table 2**, **Figure 3B**). The K_Ca_2 channel inhibitor AP30663 decreased the HR to a degree that was greater than the decrease in the TMC group.

The VERP at a paced HR of 300 bpm, was increased by the K_Ca_2 channel inhibitors AP30663 and ICA (**Table 3**, **Figure 4**). However, due to induction of VF in the 4/6 hearts during hypokalemia in the TMC group before the VERP could be established, the statistical comparison to the TMC group should be taken with a grain of salt. In the dofetilide group, VF was induced in 5/6 hearts before VERP could be established during hypokalemia. The one dofetilide heart without VF induction had a baseline VERP of 120 which was markedly higher than the mean of the other 5 dofetilide hearts at baseline (76 ± 4 ms). In this particular heart the VERP could not be measured during normokalemia since it could not be paced at a constant rate of 300 bpm. During hypokalemia, however, the VERP remained unchanged from the baseline value of 120 ms.

**Table 3:** Changes (Δdrug − baseline; Δdrug + hypo − baseline) in VERP for each group.

	Drug/vehicle Δ-VERP	n, p-value	Drug/vehicle+hypo Δ-VERP	n, p-value
TMC	−2 ± 2	6	−5 ± 5	2
3 nM Dofetilide	4 ± 7	4, 0.846	0 ± 0	1, 0.970
3 µM ICA	13 ± 3	6, 0.061	0 ± 4	5, 0.867
3 µM AP14145	8 ± 5	6, 0.308	10 ± 8	5, 0.231
3 µM AP30663	17 ± 3	6, 0.016	15 ± 2	6, 0.058

P-values refer to the comparison of Δ-values between TMC and treatment groups.

The K_Ca_2 channel inhibitor AP14145 neither changed QTcH, VERP, or HR versus baseline when compared to TMC during hypokalemia as was the case during normokalemia.

### K_Ca_2 Channel Inhibition Decreased the Risk of Ventricular Arrhythmia

Hypokalemia increased the incidence of ventricular arrhythmia in the TMC group. Further impairment of the repolarizing IKr current by dofetilide resulted in VF in five out of six animals. Two of them developed spontaneously, one before hypokalemia and one during hypokalemia. In comparison the groups receiving K_Ca_2 inhibitors only presented with VF in 1/6 (ICA and AP14145) or 0/6 (AP30663) (**Figure 3**).

**Figure 5:**
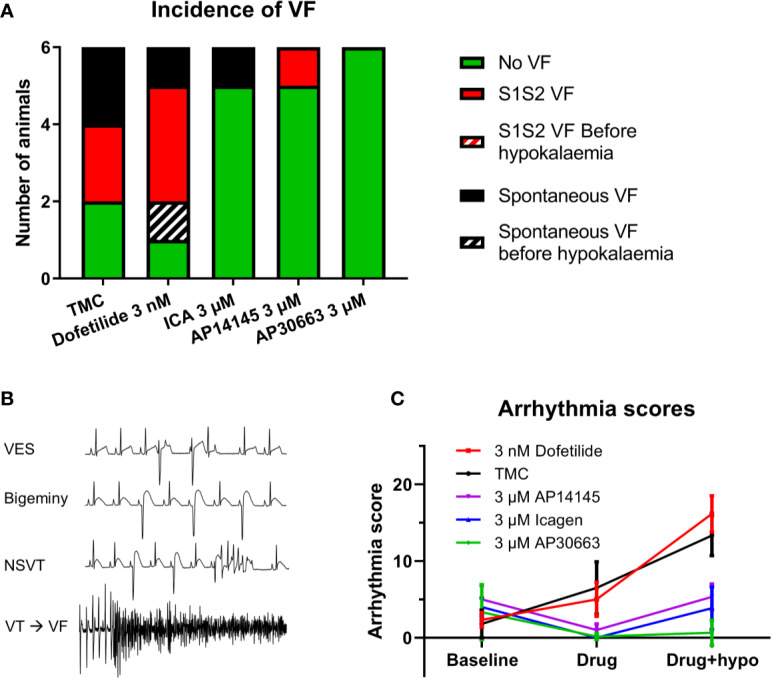
K_Ca_2 channel inhibitors reduces the incidence of ventricular arrhythmia. **(A)** Incidence of ventricular fibrillation (VF) in the five groups. **(B)** ECG examples of arrhythmias recorded during hypokalemia + 3 nM Dofetilide. **(C)** Arrhythmia score for the five groups for each perfusion period (n = 6, data are presented as mean ± SEM). ECG, electrocardiography.

The presence of VF versus no VF was matched by the arrhythmia scores in the different groups: The TMC and dofetilide were not significantly different, whereas all K_Ca_2 channel inhibitors resulted in a lower arrhythmia score, both before and during hypokalemia as compared to the TMC group (**Figure 5**, **Table 4**).

**Table 4:** Changes (Δdrug − baseline; Δdrug + hypo − baseline) in arrhythmia-score for each group.

	Drug Δ-arrhythmia score	p-Value	Drug + hypo Δ-arrhythmia score	p-Value
TMC	5 ± 4.1	NA	11.8 ± 3.2	NA
3 nM Dofetilide	2.7 ± 2.1	0.896	13.8 ± 1.8	0.937
3 µM ICA	−4 ± 1.2	0.028	−0.1 ± 2	0.002
3 µM AP14145	−4 ± 1.5	0.036	0.3 ± 3.2	0.005
3 µM AP30663	−3.2 ± 1.4	0.065	−2.7 ± 1.5	<0.001

P-values refer to the comparison of Δ-values between TMC and treatment groups. N = 6 for all groups.

## Discussion

Hypokalemia was associated with an increased incidence of ventricular arrhythmia. This was also seen in the presence of the class III anti-arrhythmic agent dofetilide. On the contrary, the incidence of ventricular arrhythmias during hypokalemia was reduced in the presence of three structurally and functionally different K_Ca_2 channel inhibitors, ICA, AP14145, and AP30663.

### Effects of Induced Hypokalemia at the Cellular Level

Hypokalemia suppresses the cardiac I_K1_, I_Kr_, and I_to_ current even though the electrical driving force for K^+^ efflux is increased. This is because lowered extracellular K^+^ concentrations speeds up the inactivation of IKr and slows the recovery from inactivation of Ito (Sanguinetti and Jurkiewicz, 1992; Firek and Giles, 1995; Yang et al., 1997) and stabilizes the blocking cations (e.g. Mg^2+^ and polyamines) in the pore of the Kir2.1 (IK1) channel [for review see (Nichols and Lopatin, 1997)], thereby decreasing the repolarizing outward K^+^ currents. Whether human K_Ca_2 channels are affected by small changes in extracellular K^+^ is unknown. Like the Kir2.1 channel, the rat K_Ca_2 channel is also inwardly rectifying partly as a result of voltage-dependent blockade by intracellular divalent cations (Soh and Park, 2001). Moreover, as for the Kir2.1 this block is increased at lower extracellular K^+^ concentration, effectively lowering the K_Ca_2 current (Soh and Park, 2001). In the present study we were unable to clearly demonstrate that outward human K_Ca_2.3 current was inhibited by lowering the extracellular K^+^ concentration from 4 mM to 2.5 mM. The discrepancy between our recordings and the study by Soh et al. is likely because we addressed the effects of much smaller changes in extracellular K^+^, but it could also stem from species or K_Ca_ subtype differences. In any case, cardiac K_Ca_2 current will not only be inhibited by hypokalemia *via* changes in cation block, but also activated by the concomitant increase in intracellular Ca^2+^ that occurs during hypokalemia. During hypokalemia the prolonged action potential duration and lowered Na^+^/Ca^2+^ activity increase intracellular Ca^2+^ concentrations (Aronsen et al., 2015; Weiss et al., 2017). It has been suggested that this increase in intracellular calcium activates ventricular K_Ca_2 channels, functioning as a protective mechanism against ventricular arrhythmia during hypokalemia (Chan et al., 2015). We addressed this hypothesis in the current study by performing experiments on isolated perfused guinea pig hearts. Lowering the extracellular K^+^ concentration to 2.5 mM resulted in minor changes of ventricular repolarization but a marked increased risk of ventricular tachycardia, which is similar to what has previously been observed in guinea pigs (Osadchii and Olesen, 2009; Osadchii et al., 2009). That hypokalemia did not produce larger changes in ventricular repolarization is likely explained by the fact that repolarization of ventricular action potential in guinea pigs is more dependent on the IKs (KV7.1/KCNE1) current as compared to IKr (KV11.1) (O’Hara and Rudy, 2012).

Application of the class III anti-arrhythmic agent dofetilide did not prevent ventricular arrhythmia. If anything, dofetilide slightly exacerbated the ventricular arrhythmia, however not to any statistically significant extent. In contrast, the K_Ca_2 channel pore blocker ICA and the negative allosteric modulators of the K_Ca_2 channel AP14145 and AP30663 significantly reduced the ventricular arrhythmia score and the incidence of VF, thereby suggesting that K_Ca_2 channel inhibition protected the ventricles from hypokalemia induced arrhythmia.

In this study we observed QTcH prolongations by AP30663 and ICA, but not with AP14145, and both also seemed to prolong the VERP. None of the K_Ca_2 channel inhibitors affected the Tp–Te interval indicating no effects on the dispersion of ventricular repolarization. In comparison, in the guinea pig heart dofetilide prolonged both QTcH and the Tp–Te interval but did not increase the VERP in the few VERP determinations that were possible to complete. Interestingly, the only heart in the dofetilide group in which VF was not induced had a markedly higher baseline VERP than the rest of the group.

Based on these few observations and the K_V_11.1 selectivity profile of ICA and AP30663 (IC_50_ > 32 µM and 4–15 µM respectively (Skibsbye et al., 2014; Bentzen et al., 2020), we speculate that the increase of the QTcH and VERP are mainly but not completely caused by ventricular K_Ca_2 inhibition.

Moreover, one could speculate that the pure K_V_11.1-driven prolongation of the QTcH and Tp–Te interval observed caused by dofetilide was pro-arrhythmic, during hypokalemia, whereas a K_Ca_2-driven prolongation of the QTcH with no effects on the Tp–Te interval appeared to protect the heart from ventricular arrhythmia. The anti-arrhythmic effects and our findings of small increases in QT and VERP (~5–20 ms) support a role of K_Ca_2 channels in the guinea pig ventricle, albeit minor under physiological conditions, which is in line with our previous studies demonstrating the expression of *KCNN2* and *KCNN3* in guinea pig ventricular tissue and ventricular anti-arrhythmic effects of K_Ca_2 channel inhibitors (Diness et al., 2015; Kirchhoff et al., 2019).

However, the effect of K_Ca_2 channel inhibition on atrial refractoriness has consistently been found to be greater than the ventricular effects and hence K_Ca_2 channels seem to show functional atrial selectivity (Diness et al., 2010; Qi et al., 2014; Diness et al., 2017; Kirchhoff et al., 2019; Bentzen et al., 2020; Diness et al., 2020). Our data during hypokalemia are different from the findings by Chan et al. who suggested that K_Ca_2 channel inhibition by the toxin apamin facilitated VF induction (Chan et al., 2015) in rabbits. Differences in species and methodology might explain this discrepancy, since they used rabbits, and optical mapping. Another difference between our study and that of Chan that could explain some of the discrepancies is the use of the pore blocking peptide apamin versus our three small molecule K_Ca_2 inhibitors of which two (AP30663 and AP14145) are negative allosteric modulators and one (ICA) is a pore blocker. Furthermore, AP14145 and AP30663 in higher concentrations (10 µM) also inhibit the late Na_V_1.5 current (Bentzen et al., 2020). Hypokalemia-induced increased intracellular Ca^2+^ has been proposed to activate Ca^2+^-calmodulin kinase thereby inducing late Na+ currents and facilitating arrhythmia (Pezhouman et al., 2015). We do not know whether this could add to the anti-arrhythmic effects and/or to a modification of the increase in QTcH observed under hypokalemia of these two drugs.

In a recent study, Tazmini et al. explored the cellular mechanisms behind ventricular and atrial arrhythmias during hypokalemia and found that hypokalemia induced Ca^2+^ overload, Ca^2+^ waves, and both early and delayed afterdepolarizations in both ventricular and in atrial cells which contain t-tubules (Tazmini et al., 2020). On the contrary, calcium was not important for development of early afterdepolarizations in atrial cardiomyocytes lacking t-tubules. In the present study we did not observe any hypokalemia induced AF episodes and based on the findings of Tazmini et al. it would be interesting to investigate if K_Ca_2 channel inhibition plays a role in this.

### Three Different K_Ca_2 Inhibitors—Similarities and Differences

We studied three structurally and functionally different K_Ca_2 channel inhibitors, the pore blocker ICA, and the two negative allosteric modulators of K_Ca_2 channels AP14145 and AP30663, which inhibit the K_Ca_2 channel by shifting the calcium activation curve to higher intracellular calcium concentrations. They all prolong the QTc (2-15 ms), and VERP but to varying degrees, with AP30663 showing the largest prolongation. Interestingly despite differences in mechanism of inhibition and effects on QTc all significantly reduced and prevented the incidence of VF during hypokalemia.

## Conclusions

Induced hypokalemia was associated with an increased risk of spontaneous or induced ventricular arrhythmia and VF, a risk that was also seen after administration of dofetilide. In contrast, the structurally and functionally different K_Ca_2 channel inhibitors, ICA, AP14145, and AP30663 decreased this risk and prevented hypokalemia-induced VF. These results support that the K_Ca_2 inhibitors, particularly AP30663, may be associated with a better safety and tolerability profile than dofetilide.

## Data Availability Statement

All datasets generated for this study are included in the article/supplementary material.

## Ethics Statement

The animal study was reviewed and approved by The Danish Ministry of Environment and Food (license No. 2014-15-0201-00390).

## Author Contributions

LA and SB contributed to the acquisition, analysis, and interpretation of data for the work. MS contributed to analysis of the data. JD and BB made the first draft of the manuscript, and contributed to the conception and design of the work as well as the analysis and interpretation of data. All authors contributed to drafting the work and revising it critically for important intellectual content

## Funding

This work was supported by the Innovation Fund Denmark, Wellcome Trust [Grant Number 100406/Z/12/Z].

## Conflict of Interest

All authors are fully or partly employed in Acesion Pharma and JD, MG, and BB are inventors of Acesion Pharma patents within the field of KCa2 channels.

The remaining authors declare that the research was conducted in the absence of any commercial or financial relationships that could be construed as a potential conflict of interest.
